# Does manufacturing specialization matter? Transfer accuracy of static surgical guides produced by general-purpose and dental-specific additive workflows: A paired in vitro study

**DOI:** 10.4317/jced.64612

**Published:** 2026-09-15

**Authors:** Mario Guerrero-Torres, Andrés Portilla-Suárez, César Augusto Ortiz-Prada, Boris Damian Jaimes-Parra, Néstor Ríos-Osorio

**Affiliations:** 1 Facultad de Odontología, Universidad Santo Tomás, Bucaramanga, Colombia; 2 School of Medicine, Universidad Autónoma de Bucaramanga, Bucaramanga, Colombia; 3 Endodontic Research Department, School of Dentistry, CES University, Medellín, Colombia; 4 Faculty of Dentistry, Institución Universitaria Visión de las Américas, Medellín, Colombia

**Keywords:** Dental implants, static computer-assisted implant surgery, surgical guide, additive manufacturing, three-dimensional printing, accuracy.

## Abstract

**Background:**

Transfer accuracy in static computer-assisted implant surgery reflects errors accumulated during imaging, planning, guide design, manufacturing, seating, and execution. This paired in vitro study compared freehand placement with static guidance produced by two additive-manufacturing workflows: A general-purpose workflow using equipment, software, and resin not indicated for dental surgical guides and a dental-specific workflow using dedicated components and surgical-guide resin.

**Materials and Methods:**

Twenty-four independent physical models were arranged in eight anatomical blocks, with one geometry-equivalent model assigned to each condition within every block: Freehand placement (FH), guided placement using a general-purpose workflow (GP), and guided placement using a dental-specific workflow (DS). Planned and achieved positions were registered three-dimensionally, and coronal, apical, and angular deviations were calculated. Friedman tests were followed by three two-sided Wilcoxon signed-rank comparisons with Holm adjustment.

**Results:**

Mean coronal deviations were 1.74 ± 0.34 mm for FH, 0.38 ± 0.23 mm for GP, and 0.40 ± 0.19 mm for DS. Corresponding apical deviations were 1.81 ± 0.34, 0.58 ± 0.25, and 0.54 ± 0.22 mm, and angular deviations were 8.69 ± 1.52°, 3.04 ± 0.50°, and 3.10 ± 0.44°, respectively. Both guided conditions produced lower deviations than FH for all three outcomes (all Holm-adjusted p=0.0234). No statistically significant differences were detected between GP and DS for coronal, apical, or angular deviation.

**Conclusions:**

Under the controlled conditions of this paired in vitro study, static guided placement reduced transfer deviation compared with freehand placement. No statistically significant difference was detected between the two guided workflows; however, the study was not designed to establish equivalence or non-inferiority.

## Introduction

The three-dimensional position of a dental implant is a critical determinant of prosthetic emergence, cleansability, and esthetic outcome [1-3]. Even modest deviations from the planned position may alter the emergence profile or reduce the available distance from adjacent teeth, cortical plates, or neurovascular structures [1-3]. 

Computer-assisted implant surgery was developed to reduce positional variability during this transfer [1-3]. Unlike freehand placement, which relies on anatomical references, visual assessment, handpiece orientation, and tactile feedback, computer-assisted approaches introduce digital or physical constraints intended to reproduce the virtual plan more consistently [1-5]. Static surgical guides can transfer the virtual plan through tooth-, mucosa-, bone-, or combination-supported designs and, when fully guided, can control osteotomy position, angulation, depth, and implant insertion [1-5].

Transfer accuracy is not determined by the guide alone. A static workflow comprises interdependent steps that include CBCT acquisition, segmentation and registration, virtual planning, guide design, additive manufacturing, post-processing, and seating [4-9]. Drill-to-guide relationships and implant insertion introduce additional mechanical tolerances [7,10-11]. Final implant-position accuracy therefore represents the cumulative effect of errors across the complete workflow rather than an isolated property of a printer or material.

Additive manufacturing may introduce further sources of geometric variation. Printing technology, resin composition, layer thickness and orientation, support configuration, washing, post-curing, sterilization, and material stiffness can influence dimensional stability and guide fit [6-12]. In parallel, guide geometry (including sleeve height and diameter, supporting length, and drill-to-guide clearance) determines the degree of residual translational and angular freedom during osteotomy preparation [7,11,12]. Thus, the manufacturing workflow is not merely a means of producing the guide; it constitutes one component of the accuracy chain through which the virtual plan is transferred to the physical surgical field.

Although fully guided protocols generally improve transfer accuracy compared with freehand placement, static guided surgery encompasses manufacturing workflows that differ in their degree of technological specialization and application-specific integration [1-5]. A dental-specific workflow may combine hardware, software, and photopolymer materials specifically developed, validated, or indicated for dental applications, including surgical-guide fabrication. In contrast, a general-purpose workflow relies on additive-manufacturing equipment, software, and photopolymer materials developed for broader applications rather than specifically for dental surgical guides. Whether this distinction in workflow specialization translates into greater geometric accuracy in the transfer of a virtual implant plan remains insufficiently established. Importantly, geometric performance under controlled experimental conditions should be distinguished from clinical suitability, including biocompatibility, sterilization compatibility, and regulatory indication. Moreover, anatomical variation may obscure workflow-related effects when techniques are evaluated across independent cases. A paired complete-block design therefore provides an appropriate framework for controlling case-specific geometry and directly comparing alternative workflows within the same underlying anatomy.

Accordingly, this study compared coronal, apical, and angular deviations from a common virtual implant plan among freehand placement, static guided placement using a general-purpose additive workflow, and static guided placement using a dental-specific additive workflow. The null hypothesis was that the three placement conditions would not differ in the distributions of coronal, apical, or angular deviations.

## Materials and Methods

*Experimental design and unit of analysis*

A controlled, paired *in vitro* experiment was conducted using a complete-block design. Eight distinct anatomical configurations (Cases 1-8), each derived from a different pre-existing, de-identified CBCT dataset, were included in the study. For each anatomical configuration, three independent, geometry-equivalent physical replicas were manufactured, one for each experimental condition: Freehand placement (FH; n=8), guided placement using a general-purpose workflow (GP; n=8), and guided placement using a dental-specific workflow (DS; n=8). Thus, the study comprised eight different underlying anatomical models, each represented by three independent physical replicas, for a total of 24 experimental models.

The experimental unit was a single implant placed in an independent physical model. Accordingly, each of the 24 osteotomies and implant placements was performed in a separate replica, ensuring that preparation of one experimental unit did not alter the mechanical conditions of another. Paired comparisons were therefore established among the three replicas belonging to the same anatomical block. 

*Geometry acquisition and virtual planning*

The experimental geometries were derived from eight pre-existing, de-identified CBCT datasets selected by convenience from the institutional archive of patients who had attended the Osseointegration Clinics at Universidad Santo Tomás in Bucaramanga, Colombia. At the time of clinical care, these patients had provided consent for the use of information from their clinical records and complementary diagnostic examinations for research purposes. Datasets were eligible when they contained complete DICOM files, provided sufficient image quality for continuous delineation of the anatomical region of interest, included an adequate field of view for segmentation and the design of a mucosa-supported surgical guide, and contained sufficient anatomical volume to accommodate the virtual planning of a 3.75 × 10 mm implant. Datasets with incomplete DICOM files, artifacts or discontinuities that prevented reproducible segmentation, or insufficient anatomical information for implant planning or guide design were excluded. No new CBCT examinations were performed for the purposes of this study.

DICOM datasets were imported into BlueSkyPlan version 5.04 (Blue Sky Bio, Grayslake, IL, USA). Thresholding and manual correction were used to isolate the anatomical region of interest and generate a three-dimensional surface, which was exported in Standard Tessellation Language (STL) format for model manufacturing and guide design. A 3.75-mm-diameter, 10-mm-long implant with an internal hexagonal connection was virtually planned for each case within the same software environment. The virtual plan defined the coronal center, apical center, longitudinal axis, and insertion depth and was maintained unchanged as the geometric reference for FH, GP, and DS within each block.

The source CBCT datasets were acquired using a GALILEOS Comfort Plus unit (Dentsply Sirona, Charlotte, NC, USA) under a standardized institutional acquisition protocol comprising an isotropic voxel size of 0.25 mm, a spherical field of view of 15.4 cm, a tube voltage of 98 kVp, a tube current of 3 mA, and an exposure time of 2 s. All datasets were exported in Digital Imaging and Communications in Medicine (DICOM) format. Following implant placement, the physical models were scanned using the same CBCT unit and acquisition parameters to maintain consistent imaging conditions between the source datasets and the post-placement scans.

*Definition of the experimental groups*

The three experimental conditions were defined according to the method used to transfer the virtual plan and the degree of specialization of the additive-manufacturing workflow (Table 1). Each guided condition was treated as a complete workflow package; no single component was experimentally varied while all remaining components were held constant.

FH (n=8) represented implant placement without a physical surgical guide, with the virtual plan available for direct consultation throughout the procedure.

GP (n=8) represented static guided implant placement using a general-purpose workflow, in which the surgical guide was manufactured using commercially available additive-manufacturing equipment, slicing software, and a photopolymer resin that were not specifically designed or indicated for dental surgical-guide applications.

DS (n=8) represented static guided implant placement using a dental-specific workflow, comprising a printer intended for dental applications, dedicated preparation software, and a manufacturer-indicated resin specifically intended for surgical-guide fabrication.

*Design and manufacturing of the models and surgical guides*

The segmented surface of each case was conditioned to generate three independent physical replicas. The model design incorporated the external anatomical contour corresponding to the mucosal support surface, a continuous internal shell defining the cortical region, and a broad internal compartment defining the medullary region. These structures were manufactured as a monolithic construction and were intended to reproduce the geometric characteristics relevant to guide support and implant placement rather than the distinct mechanical properties of soft tissue, cortical bone, or trabecular bone.

The models were manufactured using an ELEGOO Mars 5 Ultra printer (ELEGOO, Shenzhen, China) with transparent Anycubic Clear resin (Anycubic, Shenzhen, China). Printing was performed with a layer height of 0.05 mm, normal exposure of 2.8 seconds, bottom exposure of 38 seconds, five bottom layers, four transition layers, and a rest time of 0.5 seconds. After printing, the models were washed, dried, post-cured, finished, and inspected to confirm structural integrity and stability.

Mucosa-supported static surgical guides were designed for the GP and DS groups on the corresponding model surfaces. All guides were fully guided devices designed to control osteotomy position and angulation, preparation depth, and final implant insertion. The guide channel was designed to accommodate components of the CIF 4 guided-surgery system (Microdent, Barcelona, Spain). Before each procedure, complete seating and stability were verified, with particular attention to the absence of rocking or visible instability.

For GP, the STL file was prepared using CHITUBOX Basic version 1.4.1 and manufactured by masked stereolithography (MSLA) using an ELEGOO Mars 5 Ultra printer (ELEGOO, Shenzhen, China) equipped with a 405-nm light source. W200 Water-Washable Resin (eSUN, Shenzhen, China), a general-purpose photopolymer, was used. Guide-manufacturing settings included a layer thickness of 50 µm, a 45-degree build orientation relative to the Z-axis, an exposure time of 2.5 seconds per layer (with 30 seconds for bottom layers), and a medium-density tree-support strategy. The printer, software, and resin were not designed or indicated by their manufacturers for dental surgical-guide applications. Guides manufactured with W200 resin were evaluated solely as components of the GP workflow in inert models; biocompatibility, residual monomer release, sterilization compatibility, clinical mechanical behavior, and regulatory suitability were not evaluated.

For DS, the STL file was prepared using AccuWare version 3.2.1 and manufactured with an AccuFab CEL dental printer (SHINING 3D, Hangzhou, China) using the manufacturer’s validated PORTUX 3D SG material profile, a layer thickness of 50 µm, a 45-degree build orientation, and a heavy-density support strategy optimized for surgical guides. PORTUX 3D SG (New Stetic, Guarne, Colombia), a manufacturer-indicated surgical-guide photopolymer resin, was used. According to the manufacturer, PORTUX 3D SG complies with the ISO 10993 standard for biocompatibility of short-contact medical devices. The AccuFab CEL printer, AccuWare preparation software, and PORTUX 3D SG resin constituted a dental-specific workflow combination and the principal operational distinction from GP.

Following printing, the components were removed from the build platform and superficial residues were eliminated before post-curing. GP guides were cleaned and post-cured using an ELEGOO Mercury unit, whereas DS guides were cleaned and post-cured using a SHINING 3D unit. The actual protocols were as follows: GP guides were washed in water for 5 minutes, dried with compressed air, and post-cured for 10 minutes at room temperature under a 405-nm UV light; DS guides were washed in 99% Isopropyl Alcohol (IPA) for 5 minutes, dried with compressed air, and post-cured for 10 minutes at 60 °C under a 405-nm UV light source. Supports were subsequently removed and finishing was completed. Each guide was visually and manually inspected to confirm structural integrity, unrestricted passage of CIF 4 components, complete seating, and absence of rocking. Because the experiment was exclusively *in vitro*, the guides were not sterilized and were not used in patients.

*Implant placement*

Titanium Microdent implants (Microdent, Barcelona, Spain) were used in all experimental conditions. Each implant measured 3.75 mm in diameter and 10 mm in length and had an internal hexagonal connection. Within each anatomical block, the implant dimensions, virtual reference position, and planned insertion depth were kept constant. Implant placement procedures were performed by two final-year residents enrolled in the postgraduate Periodontics program who were familiar with digital implant planning and the drilling systems used in the experiment.

The 24 implant placements were distributed equally between the two operators. Each operator performed 12 placements, comprising four FH, four GP, and four DS procedures. Thus, both operators contributed equally to each experimental condition. Within every anatomical block, the three condition-specific procedures were distributed between the two operators so that both residents performed implant placements within each block. Operator assignment to the condition-specific models was randomized according to an allocation schedule established before the experimental procedures. Because the study included only two operators and four observations per operator-condition combination, it was not designed to estimate operator or operator-by-condition effects independently.

For FH placement, the operator kept the virtual implant plan visible in BlueSkyPlan version 5.04 (Blue Sky Bio, Grayslake, IL, USA) and consulted it throughout the procedure. The planned entry point, angulation, and insertion depth were reproduced using visual and anatomical references without a physical surgical guide. Osteotomy preparation was performed according to the drilling sequence specified for the 3.75 × 10 mm Microdent implant.

For GP and DS placement, the corresponding mucosa-supported surgical guide was seated on the physical model. Complete seating and stability were verified before osteotomy preparation, with particular attention to the absence of rocking or visible displacement. The CIF 4 guided-surgery sequence (Microdent, Barcelona, Spain) was then performed through the guide channel. The system components and depth stops controlled the osteotomy position, trajectory, and preparation depth. The implant was subsequently inserted through the surgical guide until the planned vertical position was achieved. Both guided protocols therefore provided guidance throughout osteotomy preparation and implant insertion.

The same standardized procedural sequence was followed for all experimental units. This sequence comprised identification of the physical model and anatomical block, verification of model integrity, confirmation of the virtual implant plan, verification of surgical-guide seating for GP and DS, sequential osteotomy preparation, removal of debris, implant insertion, and confirmation of the final vertical position. Each physical model was assigned a unique code linking the anatomical block, experimental condition, and operator. The allocation records and data from all 24 experimental units were retained throughout data acquisition and statistical analysis.

*Post-placement CBCT acquisition, three-dimensional registration, and measurements*

Following implant placement, each physical model was scanned using the same GALILEOS Comfort Plus unit (Dentsply Sirona, Charlotte, NC, USA) and the same standardized institutional acquisition protocol used for the source CBCT datasets. The reconstructed volumes were exported in Digital Imaging and Communications in Medicine (DICOM) format and imported into BlueSkyPlan version 5.04 (Blue Sky Bio, Grayslake, IL, USA). The use of the same CBCT unit and acquisition parameters for the source datasets and post-placement scans maintained consistent imaging conditions throughout the study.

Each post-placement DICOM dataset was processed using the automatic segmentation tools available in the Model Master workflow of BlueSkyPlan version 5.04. This procedure generated a three-dimensional surface model representing the external geometry of the corresponding physical model after implant placement. The resulting surface model was exported in Standard Tessellation Language (STL) format and subsequently imported into the BlueSkyPlan project containing the corresponding original anatomical model and virtual implant plan.

The post-placement STL model was registered to the corresponding original planning model using the point-based model-alignment tool available in BlueSkyPlan version 5.04. A minimum of seven corresponding reference points were selected on stable and clearly identifiable anatomical structures in the post-placement and original models. Depending on the anatomical configuration, these landmarks included incisal edges, dental pits and fissures, the anterior nasal spine when present within the model, and other distinctive anatomical features that could be consistently identified in both surface models. The implant, the osteotomy site, and anatomical regions that could have been altered by drilling or implant placement were excluded from the selection of registration landmarks. The software then calculated the spatial transformation required to superimpose the post-placement STL model on the original planning model.

The adequacy of the anatomical registration was visually assessed using the multiplanar reconstructions available in the software. The assessor examined the correspondence of the unchanged anatomical structures simultaneously in the axial, coronal, and sagittal planes while scrolling through the CBCT volume. Registration was accepted when satisfactory visual correspondence of the stable anatomical surfaces and selected landmarks was observed throughout the relevant sections, without evident spatial inconsistencies. No numerical residual registration-error threshold was used.

Whenever an inconsistency or unsatisfactory anatomical superimposition was identified, the initial registration was rejected, the corresponding anatomical landmarks were selected again, and the point-based model-alignment procedure was repeated. Registration was accepted only after the repeated procedure produced satisfactory visual superimposition of the unchanged anatomical structures. Using this verification and repetition procedure, an acceptable anatomical registration was obtained for all models included in the analysis.

After anatomical registration, the physical implant was identified directly in the post-placement CBCT volume on the basis of the characteristic radiopacity of titanium. No scan body or optical scan was used to determine the achieved implant position. A virtual implant with the same dimensions as the physical implant, 3.75 mm in diameter and 10 mm in length, was selected from the BlueSkyPlan implant library and manually positioned over the radiopaque representation of the corresponding physical implant.

Manual positioning was performed using the three-dimensional translation and rotation tools available in BlueSkyPlan. The assessor adjusted the virtual implant until its position, angulation, and central axis visually corresponded to the radiographic representation of the physical implant. The adjustment and its final verification were performed simultaneously in the axial, coronal, and sagittal multiplanar reconstruction views. Evaluation in all three orthogonal planes was used to centralize the virtual implant within the radiopaque implant image and to reduce the potential influence of metal-related scattering and boundary distortion on visual positioning. The same visualization settings and density threshold were maintained throughout the assessment of the 24 experimental units.

If the virtual implant did not show satisfactory correspondence with the radiographic representation of the physical implant in any of the three orthogonal planes, its position was rejected and the manual translation and rotation procedure was repeated. The final position was accepted only when the virtual implant was visually centered over the physical implant and showed satisfactory correspondence with its coronal extent, apical extent, and longitudinal orientation simultaneously in the axial, coronal, and sagittal views.

The virtual implant fitted to the post-placement CBCT represented the achieved implant position. Once the virtual implant had been positioned, BlueSkyPlan mathematically defined its coronal center as the geometric center of the implant platform, its apical center as the center of its apical endpoint, and its longitudinal axis as the line joining the coronal and apical centers. These reference features were therefore derived from the predefined geometry of the virtual implant and were not established through independent manual point selection.

Because the planned and achieved virtual implants were represented within the same coordinate system after registration, the evaluation tools available in BlueSkyPlan automatically calculated the positional discrepancies between them. Coronal deviation was defined as the three-dimensional Euclidean distance between the centers of the planned and achieved implants at the implant platform. Apical deviation was defined as the three-dimensional Euclidean distance between the corresponding centers of the planned and achieved implants at the apical endpoint. Angular deviation was defined as the angle between the longitudinal axes of the planned and achieved implants. Coronal and apical deviations were expressed in millimeters, whereas angular deviation was expressed in degrees.

The same CBCT segmentation, STL exportation, point-based anatomical registration, multiplanar verification, virtual-implant fitting, and measurement procedure was applied independently to the FH, GP, and DS models within each anatomical block. Each condition-specific post-placement model was registered to the corresponding original anatomical reference and virtual implant plan, allowing the achieved implant position in each experimental condition to be compared with its corresponding planned position.

All segmentation, anatomical registration, manual virtual-implant positioning, verification, and data-recording procedures were performed by a single assessor. The assessor was a dentist specialized in Periodontics with more than 10 years of clinical experience and more than five years of teaching experience in implant dentistry, osseointegration, and diagnostic imaging. The assessor was not informed of the experimental condition assigned to each coded model and did not know whether a model corresponded to the freehand (FH), general-purpose guided (GP), or dental-specific guided (DS) workflow. The same predefined procedure was applied to all 24 planned-versus-achieved comparisons.

A single definitive set of coronal, apical, and angular deviations was retained for each planned-versus-achieved implant pair. Repeated complete registration and measurement sessions were not included in the original experimental protocol because the study was primarily designed to compare the transfer accuracy of the three implant-placement workflows, and the final deviations were calculated automatically by the software after anatomical registration and manual positioning of the virtual implant. Consequently, intra-assessor and inter-assessor measurement reliability could not be quantified. This limitation was explicitly considered when interpreting the findings. The 24 complete planned-versus-achieved implant records constituted the final analytical dataset.

*Control of bias and reproducibility*

Anatomical variation was controlled through the paired complete-block design, whereby each of the eight anatomical configurations contributed one geometry-equivalent physical replica to each experimental condition. Separate physical replicas were used for FH, GP, and DS placement within every block, thereby preventing osteotomy preparation or implant placement in one condition from mechanically influencing another condition. The original virtual implant plan, implant dimensions, target position, and planned insertion depth were kept constant across the three condition-specific replicas within each anatomical block.

Implant-placement procedures were distributed between two final-year residents enrolled in the postgraduate Periodontics program. Operator assignment was randomized within the balanced experimental structure. Within each anatomical block, the three condition-specific procedures were divided between the two operators, and both operators contributed to each experimental condition across the complete experiment. Each operator performed 12 implant placements, comprising four FH, four GP, and four DS procedures. This balanced allocation reduced systematic confounding between operator and experimental condition. However, because the study included only two operators and four observations per operator-condition combination, it was not designed or powered to estimate an independent operator effect or an operator-by-condition interaction.

All implant-placement procedures were performed under controlled laboratory conditions, without patient movement, saliva, soft-tissue displacement, or limitations in clinical access or mouth opening. A standardized procedural sequence was followed for model and anatomical-block identification, verification of model integrity, confirmation of the virtual plan, guide seating and stability assessment for GP and DS, sequential osteotomy preparation, debris removal, implant insertion, and confirmation of the final vertical position.

The CBCT acquisition, segmentation, point-based anatomical registration, multiplanar verification, virtual-implant positioning, and measurement procedures were performed according to the same predefined workflow for all 24 experimental units. Post-placement imaging was performed using the same CBCT unit and acquisition parameters used for the source datasets. A minimum of seven corresponding landmarks located on stable anatomical structures was used for anatomical registration, while the implant, osteotomy site, and regions potentially altered by drilling were excluded from landmark selection. Anatomical registration was verified in the axial, coronal, and sagittal planes and was repeated whenever visual superimposition was considered unsatisfactory.

All post-placement registrations, manual virtual-implant positioning procedures, and measurement recordings were completed by a single experienced assessor. The assessor was a dentist specialized in Periodontics with more than 10 years of clinical experience and more than five years of teaching experience in implant dentistry, osseointegration, and diagnostic imaging. The experimental models were coded, and the assessor was not informed whether an individual model corresponded to the FH, GP, or DS condition. The same measurement definitions and software-based calculation procedure were applied to all planned-versus-achieved implant comparisons.

A single definitive set of measurements was obtained for each experimental unit. Repeated complete registration and measurement sessions were not included in the original experimental protocol; therefore, intra-assessor and inter-assessor measurement reliability could not be quantified. This limitation was considered explicitly when interpreting the results.

*Statistical analysis*

The dataset was screened for missing values, duplicate block-condition combinations, and group imbalance. Outcomes were summarized using means and standard deviations, medians and interquartile ranges, and minimum-maximum values. Quartiles were calculated using the empirical quantile definition with averaging at integer percentile positions. Distributional characteristics of the group observations and paired differences were assessed using Shapiro-Wilk tests and Q-Q plots.

Given the paired complete-block design, small sample, and observed departures from normality, overall comparisons used the tie-corrected Friedman test with the chi-square approximation; effect magnitude was expressed as Kendall’s W. Significant overall tests were followed by three two-sided exact Wilcoxon signed-rank comparisons. Zero differences were excluded before ranking, and tied absolute differences received average ranks at the recorded decimal precision. Exact probabilities were calculated by exhaustive sign enumeration. Holm adjustment controlled multiplicity across the three contrasts within each outcome. Paired mean differences and descriptive 95% confidence intervals were calculated using the t distribution with seven degrees of freedom, without multiplicity adjustment. Group means and t-based 95% confidence intervals were superimposed on plots of the individual within-block observations.

Sensitivity analyses added during revision included exact Friedman probabilities based on all 1,679,616 within-block label permutations and exact signed-rank comparisons using Pratt’s treatment of zero differences. Statistical significance was set at α=0.05. Analyses were performed in Python 3.12.14 using SciPy 1.18.1 and NumPy 2.5.2, with custom exhaustive-enumeration routines; figures were generated using Matplotlib 3.11.1.

## Results

All 24 implant placements in separate physical replicas were included in the analysis, comprising eight complete anatomical blocks with one FH, one GP, and one DS observation per block. No missing outcome values or duplicate block-condition combinations were identified. Descriptive statistics are presented in Table 2.

Mean coronal deviations were 1.74 ± 0.34 mm for FH, 0.38 ± 0.23 mm for GP, and 0.40 ± 0.19 mm for DS. Corresponding apical deviations were 1.81 ± 0.34, 0.58 ± 0.25, and 0.54 ± 0.22 mm, and angular deviations were 8.69 ± 1.52°, 3.04 ± 0.50°, and 3.10 ± 0.44°, respectively. Individual observations and their within-block connections are shown in Figure 1. Both guided conditions had lower deviations than FH for all three outcomes in every block; the ordering of GP and DS varied, and some paired values were identical.

The Friedman test identified overall differences among the three placement conditions for coronal deviation (χ² (2)=13.07, *P*=0.0015; Kendall’s W=0.82), apical deviation (χ² (2)=13.31, *P*=0.0013; W=0.83), and angular deviation (χ² (2)=12.45, *P*=0.0020; W=0.78).

Both guided conditions differed significantly from FH for all outcomes after Holm adjustment (adjusted *P*=0.0234 for all six contrasts; Table 3). Mean paired differences for FH minus GP were 1.36 mm coronally (95% CI, 0.98 to 1.75), 1.24 mm apically (0.94 to 1.53), and 5.65° angularly (4.30 to 7.01). Corresponding differences for FH minus DS were 1.34 mm (0.94 to 1.73), 1.28 mm (0.99 to 1.56), and 5.59° (4.15 to 7.03).

For GP minus DS, mean paired differences were -0.03 mm coronally (95% CI, -0.10 to 0.05), 0.04 mm apically (-0.06 to 0.14), and -0.06° angularly (-0.31 to 0.19). No statistically significant GP-DS difference was detected for coronal (adjusted *P*=0.6875), apical (*P*=0.4375), or angular deviation (*P*=0.6406). These nonsignificant comparisons do not establish equivalence or non-inferiority.

Sensitivity analyses added during revision yielded exact within-block permutation *P* values of 0.000400, 0.000457, and 0.000514 for the global coronal, apical, and angular comparisons, respectively. Using Pratt’s treatment of zero differences in the paired tests did not change the significance pattern; the Holm-adjusted GP-DS *P* values were 0.6875, 0.5625, and 0.7031, respectively.

## Discussion

Both guided conditions produced lower coronal, apical, and angular deviations than FH in every anatomical block. Mean deviations were approximately 0.4 mm coronally, 0.5-0.6 mm apically, and 3° angularly for the guided conditions, compared with 1.74 mm, 1.81 mm, and 8.69°, respectively, for FH (Table 2). The paired trajectories in Figure 1 show that this separation occurred across all eight blocks. Kendall's W values of 0.78-0.83 and the consistent findings in the statistical sensitivity analyses support a substantial difference among the placement conditions within this experimental setting. These findings concern geometric transfer under controlled laboratory conditions rather than clinical outcomes.

The study adds a within-anatomy comparison of complete manufacturing workflows to an established field of research. Morón-Conejo *et al*. evaluated the dimensional accuracy of guides manufactured with four printers [9], while Yang *et al*. assessed changes between the designed, printed, and post-plasma-sterilization stages [13]. Huddar *et al*. compared guided and freehand implant placement *in vitro* [14]. The present experiment complements these approaches by using separate geometry-equivalent replicas to compare GP, DS, and FH against the same virtual plan. It does not isolate printing technology or resin performance. Differences in guide support, anatomy, instrumentation, and measurement methods also limit direct comparison of absolute deviations across studies.

Final implant-position deviation integrates discrepancies introduced during imaging, planning, guide production, seating, osteotomy preparation, and insertion, together with uncertainty introduced by the postoperative assessment. This accumulation and interaction of errors can be described as tolerance stacking, but the present study did not quantify the contribution of each stage. Previous manufacturing studies have investigated the influence of printing method, material, and guide geometry [6-9,11], and post-processing has also been examined [12]. Consequently, a small final placement deviation cannot establish dimensional trueness of the printed guide, adequate polymer conversion, or stability after sterilization. Those properties require separate evaluation.

The shared CIF 4 guided-surgery system provides one plausible explanation for the small numerical GP-DS differences. Both conditions constrained osteotomy preparation and implant insertion using the same instrument system, so guide seating, channel geometry, and residual instrument movement may have contributed to their common pattern of transfer error. Experimental work with a different guided system showed that increasing lateral forces generally increased deviation and that sleeve configuration affected performance [15]. This supports the relevance of drill play without identifying its contribution in the present experiment. Neither effective instrument-to-guide clearance nor deformation under load was measured. Use of the same system therefore does not establish identical clearance, a fixed angular accuracy limit, or a predominant mechanical explanation for the approximately 3° deviations. The small sample and measurement uncertainty remain alternative explanations for the absence of detectable GP-DS differences.

The paired GP-DS estimates were small and did not consistently favor one workflow across outcomes (Table 3). Nevertheless, nonsignificant tests do not establish equivalence, non-inferiority, or interchangeability. The descriptive confidence intervals characterize uncertainty in the paired mean differences; they were not evaluated against a prespecified equivalence margin. Eight paired anatomical blocks and an analysis designed to detect differences cannot establish that any remaining difference is clinically unimportant. Equivalence would require a justified margin, adequate sample size, and a corresponding inferential design.

The geometric findings must also remain separate from material safety. W200 was a general-purpose resin without a manufacturer indication for dental surgical-guide use, and its guides were tested only on inert models. Biocompatibility, residual monomer release, sterilization compatibility, and mechanical performance under clinical conditions were not evaluated. No patient was exposed to these guides, and the present findings do not support clinical use of this material. Nor does a dental-specific designation, by itself, explain a measured geometric result. The comparison concerns the tested workflow combinations and cannot be generalized to all general-purpose or dental-specific systems.

The potential prosthetic relevance of the guided-FH difference involves both platform position and implant orientation. Clinical research has emphasized the importance of these parameters for reproducing the intended restorative position [16]. In the present study, the lower coronal deviations with GP and DS indicate closer reproduction of the planned platform location, while their lower angular deviations indicate closer reproduction of the planned axis. These differences may help preserve the intended emergence and screw-access trajectory, but no restorations were fabricated or evaluated. The restorative consequence of a deviation depends on its direction, crown geometry, planned implant position, and available components. Thus, the observed angular means cannot determine whether a case would permit direct screw retention or require angled components or cementation, and approximately 3° should not be interpreted as a universal prosthetic acceptability threshold.

Apical deviation provides a complementary geometric perspective. Mean apical deviation exceeded mean coronal deviation in all conditions, although the difference between these means was small for FH. For the 3.75 × 10 mm implants used here, displacement at the apex reflects the combined effects of translation and rotation, including their directions. Group mean angular deviation cannot be converted directly into the observed mean apical deviation, and scalar coronal and angular errors cannot simply be added to explain it. The data therefore support closer reproduction of the planned position with guidance, but do not quantify anatomical safety margins or predict injury. Clinical planning still requires assessment of the actual distance to adjacent structures.

The complete-block design controlled case-related anatomy, and separate replicas prevented one osteotomy from mechanically affecting another experimental condition. Both residents contributed equally to each condition, with four FH, four GP, and four DS placements per operator, reducing systematic confounding between operator and technique. However, balancing allocation does not eliminate operator variability. With only two final-year residents, the study could not estimate an independent operator effect or an operator-by-condition interaction. The FH findings also relate specifically to placement with the virtual plan available for consultation, rather than to an unplanned procedure. Generalization to operators with different levels of experience requires further investigation.

The *in vitro* model imposes important biomechanical limitations. The monolithic Anycubic Clear construction reproduced the external support contour and the engineered cortical-medullary organization, but not the distinct mechanical behavior of biological mucosa, cortical bone, and trabecular bone. The nominally mucosa-supported guides therefore rested on a rigid representation of that surface, without physiological tissue compression or displacement. Shared model construction improved consistency across conditions, but did not establish that drilling resistance or guide stability reproduced clinical conditions. Saliva, patient movement, and restrictions in access or mouth opening were also absent.

Surface inclination and drilling forces offer a specific reason to be cautious when extrapolating these results. Ou *et al*. found that varying the inclination of artificial bone surfaces affected placement accuracy even with a static fully guided protocol [17]. Together with the evidence on lateral forces [15], this indicates that guidance constrains the drilling trajectory without eliminating deviation associated with instrument loading and the entry surface. Drill deflection, entry-point slipping, and local resistance were not measured in the present models, and their behavior cannot be assumed to match that of heterogeneous living bone. These factors could alter both guided and FH performance in patients; the present experiment cannot determine whether their between-condition difference would become larger or smaller clinically.

Sample size and anatomical selection further limit generalizability. Although 24 independent replicas were used, the number of distinct anatomical configurations was eight, selected by convenience from an institutional archive. Pairing improved control of anatomical variability but did not increase the diversity of the source anatomy. The consistent guided-FH ordering across blocks supports the observed within-sample pattern, but cannot establish its magnitude across different ridge morphologies, support areas, or implant sites. The results also do not establish performance for other implant dimensions or guided systems.

Measurement uncertainty is a separate limitation from placement variability. A single experienced periodontist, blinded to group assignment, applied a standardized workflow using at least seven landmarks on unchanged anatomy, exclusion of the implant and osteotomy region from landmark selection, and multiplanar verification. These measures were intended to reduce inconsistent registration and group-related assessment bias. However, anatomical alignment and manual fitting of the virtual implant were accepted visually, without a numerical residual-error threshold. Repeating an unsatisfactory fit during that process constituted quality control, not an independent repeatability assessment. Because only one definitive measurement set was retained per pair, intra-assessor and inter-assessor reliability remain unquantified.

Automatic calculation of deviations does not remove uncertainty in the manually fitted implant position from which they are calculated. In an experimental comparison of postoperative assessment methods, Oh and Lee found greater deviations and interoperator variability with manual CBCT-based approaches than with automatic matching using a scan abutment [18]. Their protocol was not identical to the present BlueSkyPlan workflow, so their findings neither validate nor quantify the error of our measurements. They nevertheless demonstrate why examiner experience and software-generated coordinates cannot substitute for direct reliability assessment. Voxel sampling at 0.25 mm, segmentation, anatomical registration, and metal-related boundary distortion may each influence localization. No independent metrological reference was used to separate these contributions. This unresolved uncertainty is particularly relevant to the small GP-DS differences, and statistical sensitivity analyses cannot correct it.

Further work should distinguish manufacturing error, surgical transfer error, and measurement error. Direct assessment of guide dimensions, seating, clearance, and deformation could test the proposed role of shared mechanics. Repeated complete registration and implant-localization sessions, together with an independent positional reference, would address repeatability and trueness. Factorial experiments could separate printer, resin, software, and post-processing effects, while larger samples could examine operator variability and biomechanically more realistic conditions. These are priorities for subsequent research, rather than evidence supplied by the current experiment.

Overall, static guidance reduced measured transfer deviation relative to FH across the eight paired anatomical blocks. The study did not detect a difference between GP and DS, but cannot establish equivalence or identify the component responsible for the observed pattern. Its contribution is a controlled workflow-level comparison, with explicit limits arising from the model, sample, and unquantified measurement reliability. The results do not establish clinical suitability of the non-dental resin.

Finally, the clinical relevance of transfer accuracy extends beyond geometric agreement. Ríos-Osorio *et al*. describe the importance of peri-implant soft-tissue characteristics and their interactions with implant-prosthetic surfaces for tissue integration and peri-implant health [19]. Complementarily, Mutis Gómez *et al*. identify post-processing quality control, operator training, and economic feasibility as important considerations when integrating additive manufacturing into dental workflows [20]. These perspectives reinforce the distinction between reproducing a planned implant position and validating a workflow for clinical use. The present study assessed geometric transfer under controlled laboratory conditions, but did not evaluate tissue responses, long-term peri-implant outcomes, or cost-effectiveness. Accordingly, its findings should inform further workflow evaluation without being interpreted as evidence of biological safety, clinical interchangeability, or economic superiority.

## Conclusions

Within the limitations of this paired *in vitro* study, both guided conditions produced lower coronal, apical, and angular deviations from the virtual plan than FH, and this pattern was observed in all eight anatomical blocks. No statistically significant difference was detected between GP and DS; however, the study was not designed to establish equivalence or non-inferiority. Because the guided conditions comprised different complete manufacturing packages, the contributions of individual printers, resins, software, and post-processing procedures could not be isolated. The geometric findings observed for the GP workflow do not establish the biocompatibility, regulatory suitability, or clinical safety of the non-dental resin included in that workflow and do not support its use in patients. 

## Figures and Tables

**Table 1:** Table 1: Operational definition of the experimental groups.

**Group**	**Meaning**	**n**	**Operational definition**
FH	Freehand	8	Osteotomy and implant insertion were performed without a physical guide. The operator kept the BlueSkyPlan 5.04 plan visible and reproduced entry point, angulation, and depth using visual references.
GP	General-purpose guided workflow	8	The mucosa-supported guide was manufactured using an ELEGOO Mars 5 Ultra printer, general-purpose slicing software, and eSUN W200 Water-Washable resin, none indicated for dental surgical-guide use. The fully guided CIF 4 protocol controlled osteotomy, depth, and implant insertion.
DS	Dental-specific guided workflow	8	The mucosa-supported guide was manufactured using an AccuFab CEL dental printer, AccuWare software, and PORTUX 3D SG surgical-guide resin. The fully guided CIF 4 protocol controlled osteotomy, depth, and implant insertion.

FH: Freehand placement; GP: Guided implant placement using a general-purpose workflow; DS: Guided implant placement using a dental-specific workflow. Each case contributed one observation to each group.

**Table 2:** Table 2: Deviations from the planned implant position by placement condition.

**Outcome**	**FH (n=8)**	**GP (n=8)**	**DS (n=8)**
Coronal deviation (mm)	1.74 ± 0.34 1.70 [1.50, 2.10] 1.20-2.10	0.38 ± 0.23 0.30 [0.25, 0.50] 0.10-0.80	0.40 ± 0.19 0.30 [0.30, 0.55] 0.20-0.70
Apical deviation (mm)	1.81 ± 0.34 1.70 [1.60, 2.00] 1.40-2.50	0.58 ± 0.25 0.50 [0.40, 0.75] 0.30-1.00	0.54 ± 0.22 0.50 [0.35, 0.70] 0.30-0.90
Angular deviation (°)	8.69 ± 1.52 8.89 [7.60, 9.93] 6.30-10.40	3.04 ± 0.50 3.00 [2.65, 3.50] 2.30-3.70	3.10 ± 0.44 3.05 [2.80, 3.25] 2.60-4.00

Each anatomical block contributed one observation per condition. Within each cell, the first line shows mean ± standard deviation, the second shows median [Q1, Q3], and the third shows minimum-maximum. Q1 and Q3 are the 25th and 75th percentiles, calculated using the empirical quantile definition with averaging at integer percentile positions. FH: Freehand placement; GP: Guided placement using a general-purpose additive workflow; DS: Guided placement using a dental-specific additive workflow.

**Table 3:** Table 3: Paired differences and exact comparisons between placement conditions.

**Outcome**	**Contrast**	**Mean difference (95% CI)**	n*	**W**	**Adjusted ** *P*
Coronal (mm)	FH - GP	1.36 (0.98 to 1.75)	8	0.0	0.0234
	FH - DS	1.34 (0.94 to 1.73)	8	0.0	0.0234
	GP - DS	-0.03 (-0.10 to 0.05)	6	7.0	0.6875
Apical (mm)	FH - GP	1.24 (0.94 to 1.53)	8	0.0	0.0234
	FH - DS	1.28 (0.99 to 1.56)	8	0.0	0.0234
	GP - DS	0.04 (-0.06 to 0.14)	5	4.0	0.4375
Angular (°)	FH - GP	5.65 (4.30 to 7.01)	8	0.0	0.0234
	FH - DS	5.59 (4.15 to 7.03)	8	0.0	0.0234
	GP - DS	-0.06 (-0.31 to 0.19)	7	10.5	0.6406

The mean difference is the first condition minus the second. Mean differences and confidence intervals use all eight paired observations for each contrast. CIs were calculated from the paired differences using the t distribution with seven degrees of freedom; they are descriptive and are not adjusted for multiple comparisons. n*: Number of nonzero paired differences included in the signed-rank test. Zero differences were excluded before ranking; Tied absolute differences received average ranks at the recorded decimal precision. W is the smaller of the positive and negative rank sums, distinct from Kendall’s W reported for the overall comparison. Exact two-sided probabilities were obtained from all possible sign assignments and adjusted using Holm’s procedure across the three contrasts within each outcome. CI: Confidence interval; FH: Freehand; GP: General-purpose guided workflow; DS: Dental-specific guided workflow.

**Figure 1:**
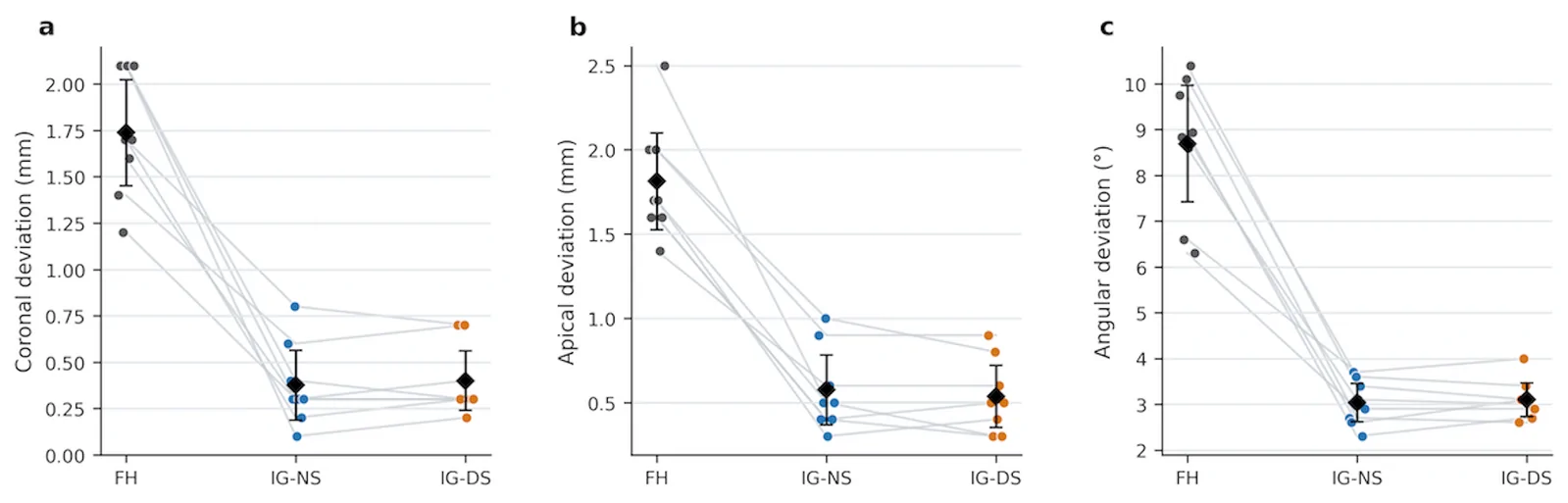
Fig. 1: Deviations from the planned implant position across eight anatomical blocks. a: Coronal deviation in millimeters; b: Apical deviation in millimeters; c: Angular deviation in degrees. Each symbol represents one implant placement in a separate physical replica. Light-gray lines connect observations from the three geometry-equivalent replicas within the same anatomical block; they do not represent repeated placements in a single model. Open circles, squares, and triangles denote FH, GP, and DS, respectively. Black diamonds and vertical error bars indicate group means and descriptive 95% confidence intervals calculated using the t distribution. Horizontal offsets separate overlapping observations. FH: Freehand placement; GP: Guided placement using a general-purpose additive workflow; DS: Guided placement using a dental-specific additive workflow.

## Data Availability

The de-identified numerical dataset underlying the reported analyses is available from the corresponding author upon reasonable request. Source CBCT datasets remain subject to institutional authorization and applicable data-protection restrictions.
